# Supplementary material for: Fast and light-efficient remote focusing for volumetric voltage imaging
Source: Nat Commun. 2024 Nov 5;15:9555. doi: 10.1038/s41467-024-53685-5 (PMC11538346; doi:10.1038/s41467-024-53685-5)
Supplement: Supplementary file 1 — Supplementary Information [file 41467_2024_53685_MOESM1_ESM.pdf]

Fast and light efficient remote focusing for volumetric voltage imaging – Supplement 1

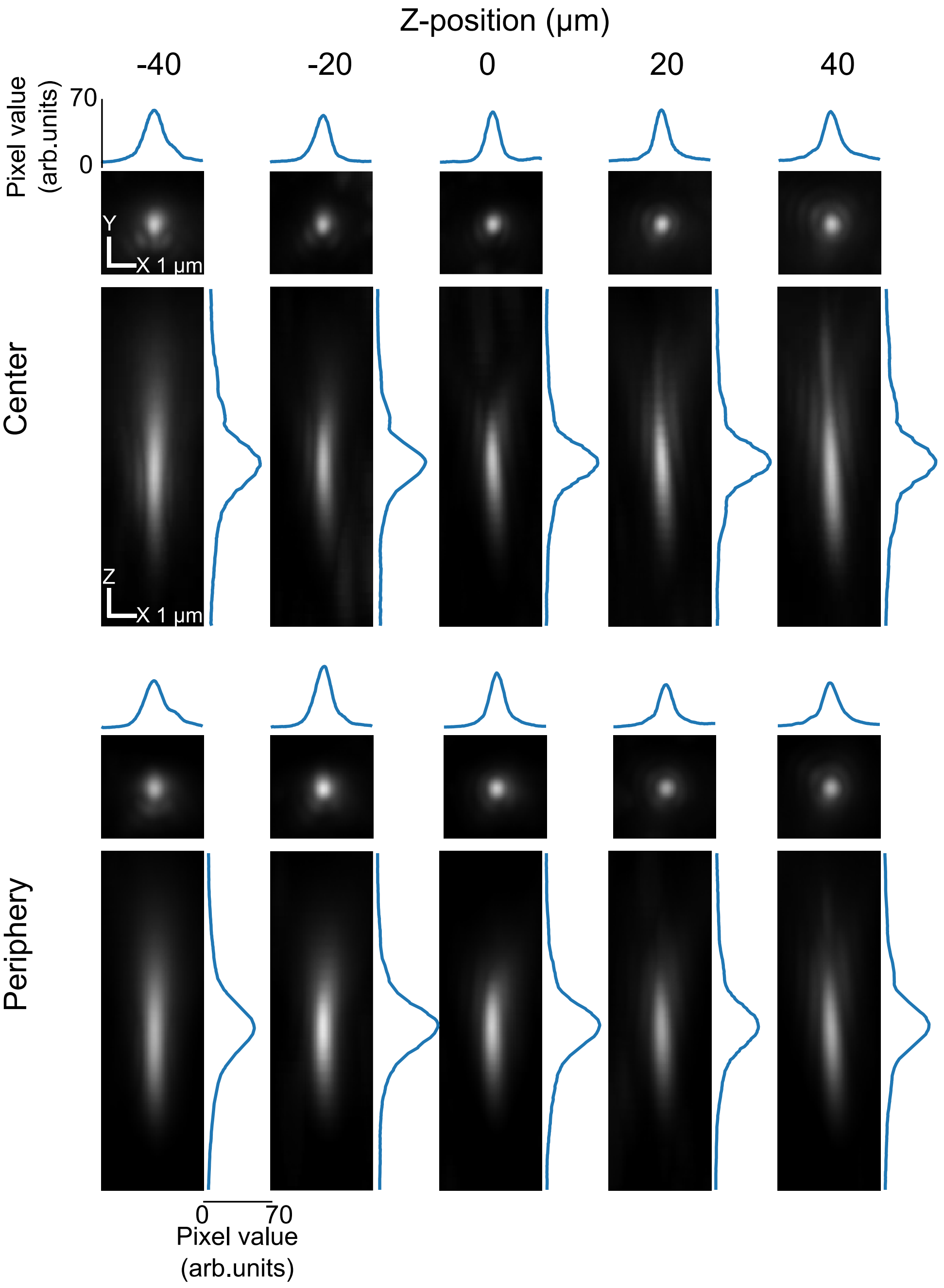

Fig. S1. Measured point spread functions at the center and periphery: Details of measured point spread functions across a z-range of 80 µm. Each PSF represents the average image of 26-127 0.1 µm diameter fluorescent beads. The plotted curves next to each PSF are the cross section through the center of the image along the x and z axis respectively.

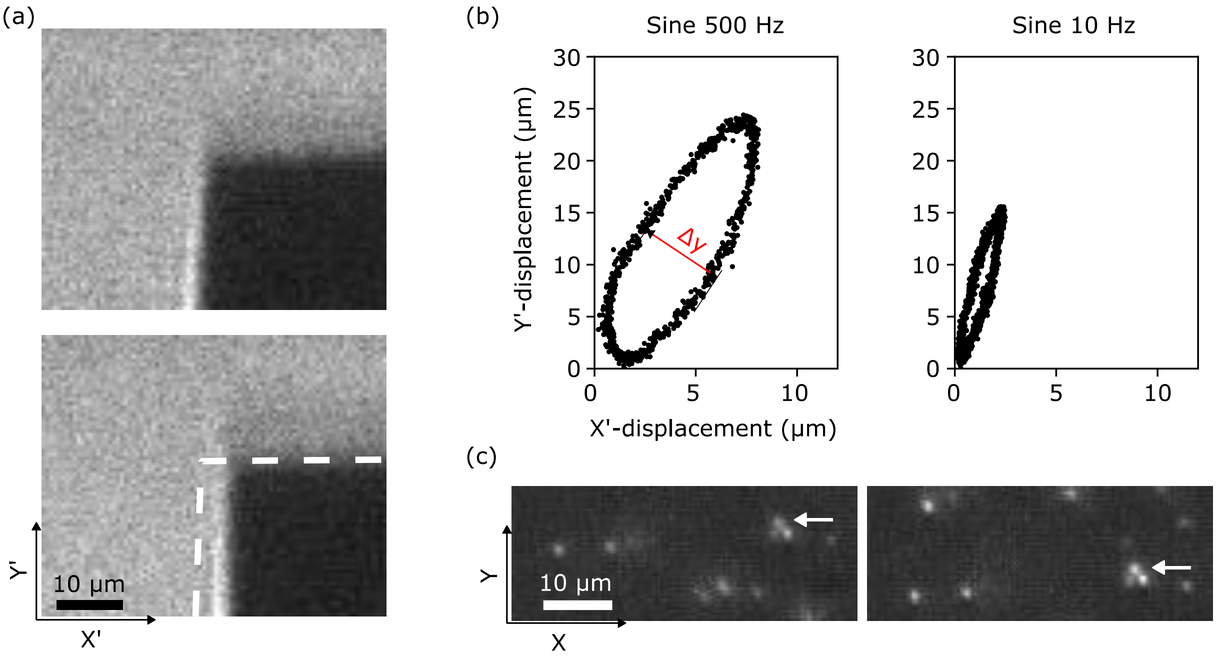

Fig. S2. lateral image shift during refocusing: (a) Still frames of a close-up high-speed video of the edge of the remote retroreflector during high-speed refocusing. The first frame shows the position during the up-stroke, the second frame the position half a period later during the down-stroke. For the same y'-position, the retroreflector is shifted along x'. (b) Quantification of the lateral shift at two different drive frequencies. Higher frequencies lead to a larger shift in x'. (c) Average fluorescence of fluorescent beads during high-speed refocusing showing the same position in the volume during the up-stroke and the down-stroke. The result of the movement quantified in b is a translation of the image along the y axis. Even though the retroreflector shifts around along both dimensions, only the y-component leads to a translation of the actual image since this the axis perpendicular to which the image is folded in the remote focusing plane.

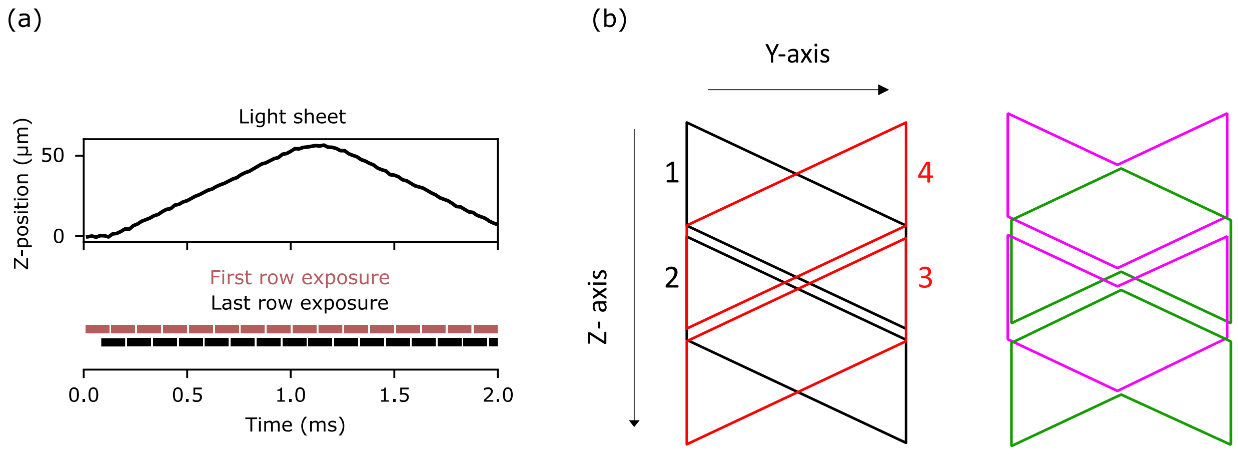

Fig. S3. Rolling shutter during fast acquisition: (a) Light sheet position during one period of volume scanning and the exposure timing of the first and last row in the 144 row image. Due to the rolling shutter of the sCMOS camera, the last row starts exposing 76.25 µs after the first row which results in the image being tilted in the recorded volume. (b) Workflow of combining tilted image planes from up- and down-stroke into one consecutive z-stack. The combination of the first half of the first frame of the down-stroke (frame 1 in the schematic) with the second half of the last frame of the up-stroke (frame 4) is combined to become the first image in the z-stack (top purple frame). The second image in the z-stack (top green frame) is combined from the first half of the last frame on the up-stroke (frame 4) and the second half of the first frame of the down-stroke (frame 1).

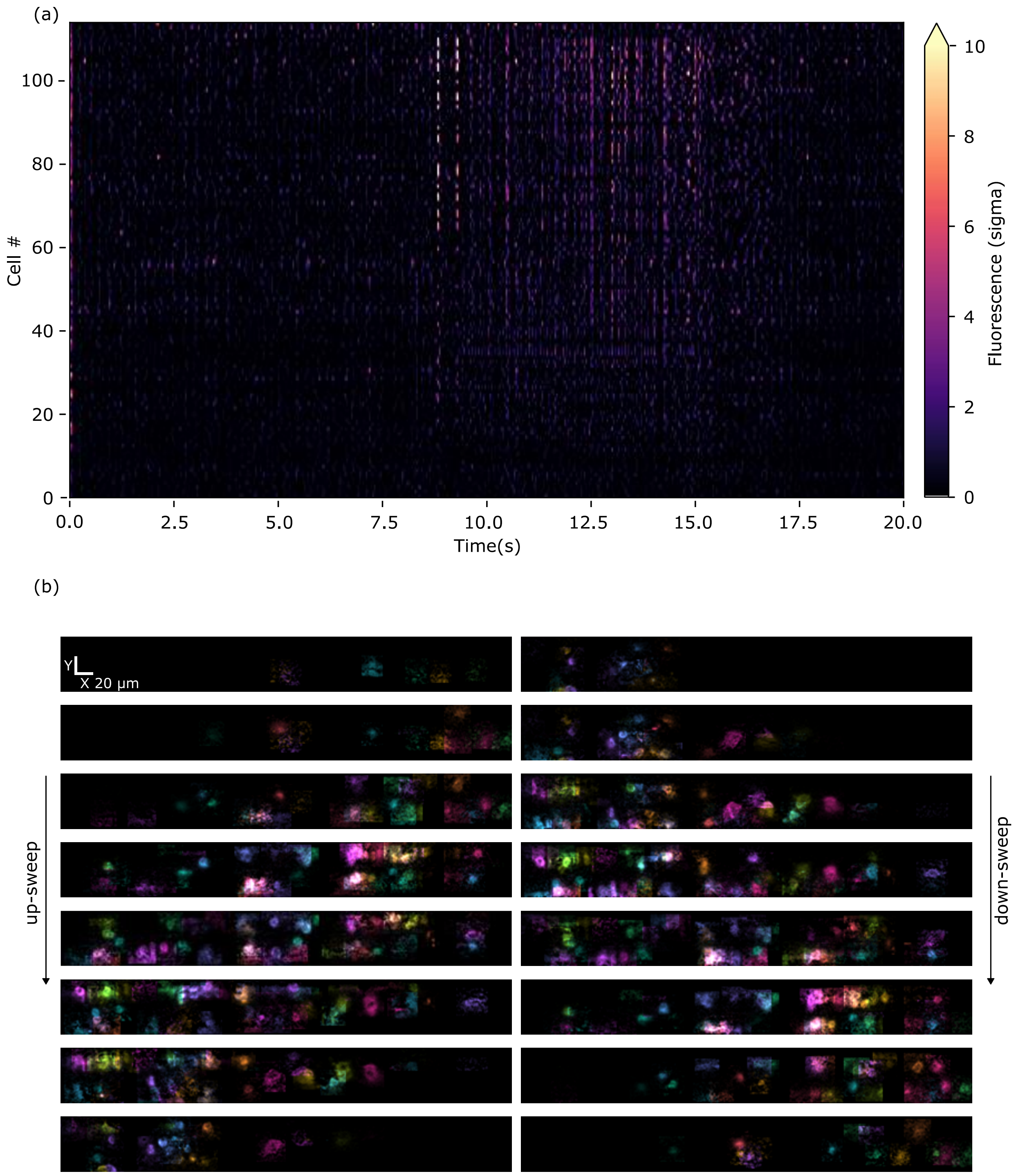

Fig. S4. Parallel recording of >100 neurons in a spinal cord volume: (a) Activity of 114 neurons in the volume form Fig 3, recorded over 20s. Traces were manually inspected to exclude those that did not show obvious neural activity and close by ROIs that were obviously overlapping. (b) Spatial footprints of the traces shown in a. All 16 frames of one volume sweep are shown with most footprints covering at least two consecutive frames during the up- and the down-sweep. Footprints are assigned random colors for visualization. Z-depth of the volume 50 µm.

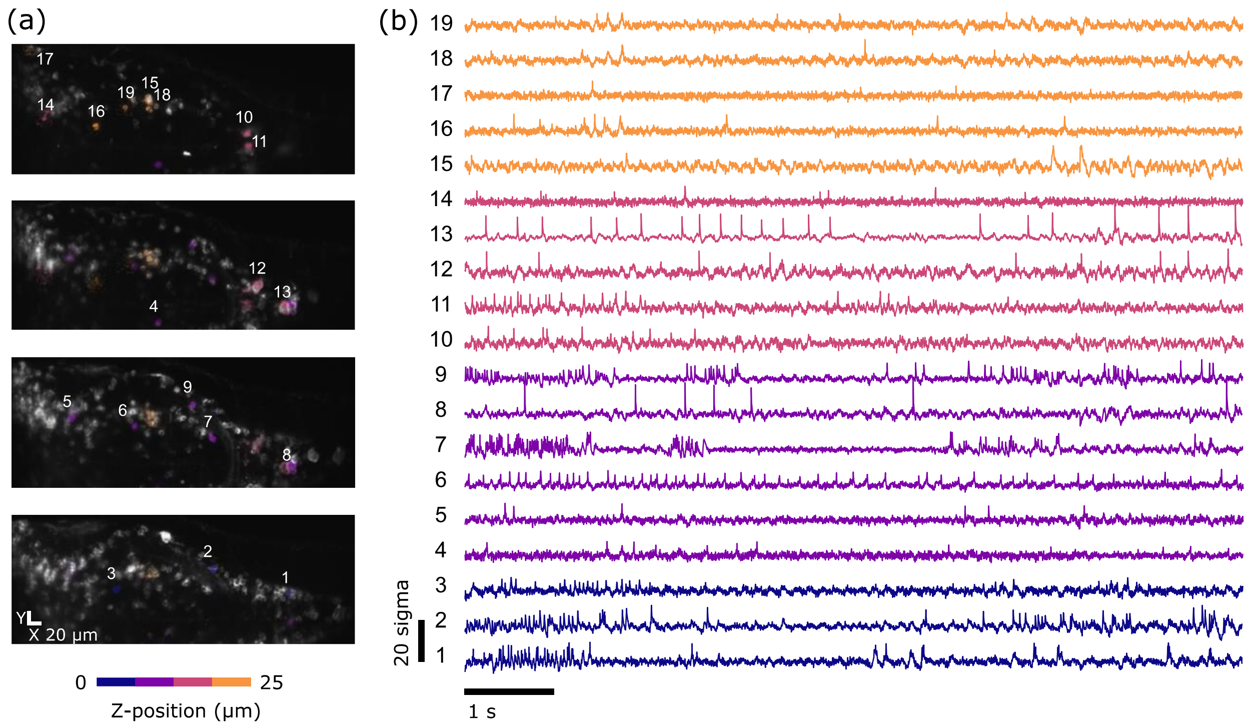

Fig. S5. Volumetric imaging from hindbrain with increased FOV: (a) Average fluorescence and footprints of selected neurons of 4 planes of lateral hindbrain of a 4 dpf *Tg(HuC:Gal4; UAS:Voltron2-ST)* zebrafish larva (x = 390 µm, y = 92 um, z = 25 µm). Footprints are visible over several z-sections but numbered only once. (b) Fluorescent traces corresponding to the spatial footprints shown in b (z-scored), ordered by z-position. Color of footprints in a and traces in b denote z-position in the volume. Representative example of an experiment with 4 fish.

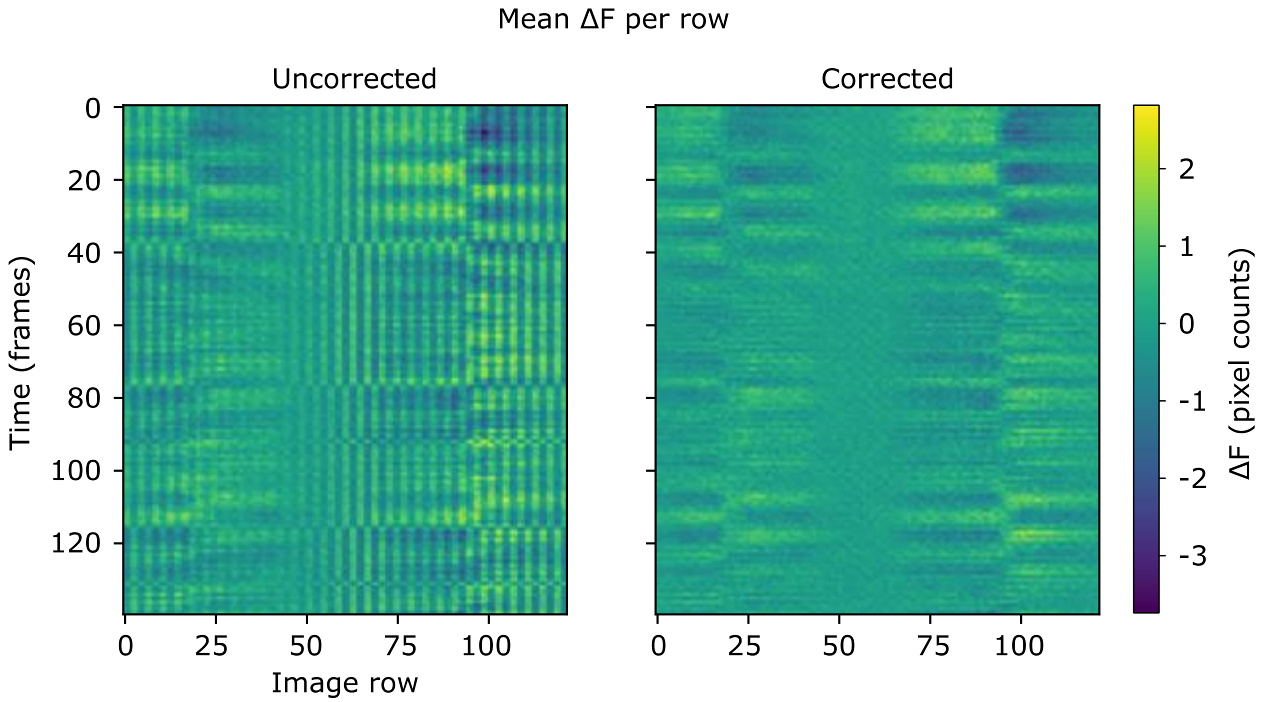

Fig. S6. Exposure time correction due to frame trigger and camera clock mismatch: ΔF averaged per row for 140 frames from an arbitrary plane of a 500 volumes/s recording. The uncorrected data shows a changing offset that is different for each row adding noise to the recording.
