# Supplementary material for: Fast and light-efficient remote focusing for volumetric voltage imaging
Source: Nat Commun. 2024 Nov 5;15:9555. doi: 10.1038/s41467-024-53685-5 (PMC11538346; doi:10.1038/s41467-024-53685-5)
Supplement: Supplementary file 2 — Description of Additional Supplementary Files [file 41467_2024_53685_MOESM2_ESM.pdf]

Description of Additional Supplementary Files

Supplementary Movie 1. Sample video of volumetric voltage imaging data. 1s volumetric data (8 planes) spanning the entire volume of the spinal cord. Same dataset as displayed in Fig. 3 and Fig. S4. Gray: Average fluorescent signal over the entire recording. Overlay: Spatial footprints modulated in time by the z-scored $\Delta F/F$ of all 114 selected neurons. Overlay adjusted for contrast.
